# Consumer awareness campaign to reduce household food waste based on structural equation behavior modeling in Hungary

**DOI:** 10.1007/s11356-020-09047-x

**Published:** 2020-06-25

**Authors:** Dávid Szakos, Barbara Szabó-Bódi, Gyula Kasza

**Affiliations:** 1 National Food Chain Safety Office, Budapest, H-1024 Hungary; 2 University of Veterinary Medicine, Budapest, Hungary

**Keywords:** Household food waste, Consumer behavior, Attitude model, PLS-SEM, Awareness raising, Consumer campaign

## Abstract

The aim of this study is to explore behavioral patterns behind household food waste with partial least square structural equation modeling (PLS-SEM). Results are based on a quantitative consumer survey with personal interviews. Sample (*n* = 1002) is representative of the adult population of Hungary in regard to age, sex, and geographical distribution. Statistical analysis included descriptive tests, variance analysis, principal component analysis, factor analysis, and PLS-SEM modeling. Based on multivariate tests, income, age, education, residence, and region were identified as the most influential socio-demographical factors of food wastage. Within the framework of the attitude model, the first PLS-SEM model (normative model) validated that all three—cognitive, affective, and conative—attitude components have an effect on food wastage behavior, but the conative component revealed to be the most important one. This underlines the importance of childhood education and awareness raising to shape routines and behavioral patterns with proper messages and impulses. Based on the second PLS-SEM model (explicative model), *cooking too much food* was identified as the most prominent pattern that influences food wastage. Contrary to anticipations, *unplanned food purchase* represented only minor significance. The results provided behavioral insights to a national level food waste prevention campaign in Hungary, called *Wasteless* (*Maradék nélkül*). This campaign plays an important role to meet the requirements of new EU legislation on food waste and the recommendations of EU Platform on Food Losses and Food Waste.

## Introduction

Food waste is generated in every stage of the food chain, from agricultural production to households. In economically developed countries, the most significant part of food waste derives from the households, which equals to 53% of total food waste in Europe (FAO 2011; FUSIONS 2016). There have been many publications investigating different food waste treatment methods, such as composting, anaerobic digestion, fermentation, or other industrial uses (Zhang et al. 2018). However, determining of the factors behind and prevention of food waste has to be equally important parts of the research activity.

While the effect of some socio-demographic factors, such as income and household type on food waste production seems to be well-known (Schneider and Obersteiner 2007; Evans 2011), many influencing factors hide silently behind routine household activities as attitude elements (Frohnmaier et al. 2015). In this study, factors determining the amount of household food waste are introduced on the basis of an attitude model, suggested initially by Allport (1935), who established the multidimensional interpretation of attitude. According to his attitude model theory, attitude consists of cognitive, affective, and conative components, which are consistent in affecting the individual’s general behavior. Firstly, cognitive component influences the attitude through conscious and logical thinking. Secondly, affective component is connected to feelings. Thirdly, conative component has an effect on the attitude by habits and not conscious actions. Therefore, based on the multidimensional model of attitudes, every attitude is constructed of the previously listed three components. This view persisted throughout the twentieth century and provided theoretical support for extensive research (Ostrom 1969; Fishbein and Ajzen 1977; Greenwald 1989; Eagly et al. 1994), and can still be used in the light of recent developments of the attitude theory (Ajzen and Fishbein 2005; Agapito et al. 2013). Considering the fact that household food waste depends heavily on socio-demographic background, we also examine the role of these factors in our study.

In recent times, several studies appeared that approached consumer level food waste production from the aspect of attitudes and behavioral elements (Evans 2012; Koivupuro et al. 2012; Williams et al. 2012; Ganglbauer et al. 2013; Abeliotis et al. 2014; Graham-Rowe et al. 2014; Parizeau et al. 2015; Mallinson et al. 2016; Djekic et al. 2019). Besides attitudes, lack of knowledge is also a significant problem, and even informed consumers have trouble with implementing their knowledge during their everyday activities (Porpino et al. 2016). Researchers tend to agree that changing consumer behavior by raising awareness and putting routine activities in a different light are key factors in enhancing the sustainability of the food chain (Evans 2011; Farr Wharton et al. 2014; Stancu et al. 2016; Stangherlin and Barcellos 2018). The Hungarian National Food Chain Safety Office (NFCSO) started its household-level food waste prevention program, called *Wasteless* (*Maradék nélkül*) in 2016 based upon these principles. This communication program has been supported by research elements from the beginning. To get an accurate picture of the quantity of household food waste, a measurement was conducted in 2016 with the standardized EU methodology (FUSIONS 2014), which is now integrated into the Supplementing Directive of EU (EC 2019). The survey was based on physical weighing of food waste. As a result of this research, we found that an average Hungarian consumer generates 68 kg of food waste annually, of which 49% could have been avoidable (Szabó-Bódi et al. 2018). A similar measurement was conducted in a recent study in Greece (Abeliotis et al. 2019). They revealed that the estimated per capita food waste generation in their country is 76.1 kg annually. Concerning our research, the proportion of certain meals and food types in the wastage was also measured, as well as the frequency of certain valorization methods (such as composting and animal feed). These pieces of information were essential in defining target indicators in food waste reduction, but did not explain the roots of consumer behavior that resulted in an excessive amount of food waste. For a better understanding, we formulated two hypotheses based on key findings from recent literature (Mondéjar-Jiménez et al. 2016) and tested them in another consumer research that used quantitative methodology:

- H1: Apart from demographical factors, household food waste is significantly affected by attitudes, of which the conative component is the most prominent.
- H2: Unplanned food shopping practices contribute to household food waste to a lesser extent than the preparation of unreasonable amount of food.

With the last modification of the EU legislation on food waste, from now on, all EU Member States have to conduct awareness campaigns on food waste prevention (EC 2018, 2019). Recently, the EU Platform on Food Losses and Food Waste made recommendation that in implementing national strategies to prevent food waste, Member States should make full use of latest findings of behavioral science research and integrate food loss and waste in education (EU Platform on Food Losses and Food Waste 2019).

The overall aim of the present research is to reveal consumers’ behavioral patterns regarding household food waste generation applying PLS-SEM model. The results provided with appropriate input in developing *Wasteless*, a Hungarian awareness raising campaign dealing with household food waste prevention. The campaign plays an important role to meet the requirements of new EU legislation.

## Methodology

For data collection, personal, face-to-face interviews were used (pen-and-paper type), with respect to generally accepted preconditions (Babbie 2014; Lourenço et al. 2016). During the designing of the questionnaire, we relied on experience from previous food waste-related studies (Cox et al. 2010; Stefan et al. 2013; Jörissen et al. 2015). We applied both open and close ended, as well as Likert-scale rating questions. The questions covered the following food waste-related topics:

- Consumer awareness of food wastage
- Consumer attitudes related to food wastage
- Consumers’ level of awareness regarding food labeling and storing (true or false questions about best before and use by dates, proper temperature of the fridge, storing of different types of food)
- The most frequently discarded food types in households

Interviews were conducted from 19th of November to 9th of December in 2016 (*n* = 1002). In terms of age, sex, and geographical distribution, the sample is representative of the adult population of Hungary, based on the latest population census result available at the time of the data collection (HCSO 2012). Representativeness of the sample was ensured by setting quotas for the demographic factors, which were followed during sample collection. The sampling of the respondents was conducted near railway stations and main traffic junctions of Budapest and eight other cities of Hungary. In the sampling area, the appointed interviewers randomly chose potential respondents (paying attention to the quotas set for each demographic factor that served as the basis for representativeness) and asked for an interview. The interviews were conducted in the Hungarian language. The composition of the sample compared to the adult Hungarian population is presented in Table 1, other demographical characteristics are shown in Table 2.

**Table 1:** Composition and representativeness of the sample

		Respondents	Hungarian population
Sex	Female	53%	53%
	Male	47%	47%
Age	18–29	18%	16%
	30–39	20%	20%
	40–59	34%	35%
	60+	27%	29%
Habitation	Central Hungary	30%	30%
	Central Transdanubia	11%	11%
	Western Transdanubia	10%	10%
	Southern Transdanubia	9%	9%
	Northern Hungary	12%	12%
	Northern Great Plain	15%	15%
	Southern Great Plain	13%	13%

**Table 2:** Other demographical characteristics

		Respondents
Habitation type	Village	14%
	Town	63%
	Capital (Budapest)	22%
Education level	Primary school	6%
	Vocational school	9%
	High school	31%
	University	54%
Income level	Low	18%
	Average	62%
	High	19%
Presence of children (under 6 years old)	Yes	24%
	No	76%

For descriptive and multivariate statistical analysis, IBM SPSS Statistics 22.0 was used. For Structural Equation Modeling (SEM), we applied the Smart PLS software that is capable of second-generation data analysis. With the SEM method, two operations can be done simultaneously: a factor analysis that combines the influencing variables into new, so-called latent variables, and a regression analysis that examines the relationship between these newly created latent variables (Wong 2013). This method is used to quantify the separate and cumulative effect of influencing variables on a target variable. It has recently been applied in other food waste and sustainability-related studies (Hameed et al. 2019; Grasso et al. 2019). Two types of SEM modeling are used widely: the covariance-based (CB) and the variance-based partial least square (PLS) method. For the analysis of the gathered data, the PLS method was more appropriate considering that it handles ordinal scales, such as Likert scales (which are often used in social science) and does not require normal distribution (Haenlein and Kaplan 2004). PLS-SEM model consists of an outer model, which demonstrates the connection between the observed variables and the constructed latent structures, and an inner model describes the regression routes between the latent variables (Henseler et al. 2012). The structure of the latent variables can be either reflective or formative. In case of reflective structures, there is a strong correlation between the explanatory variables, while in case of formative structures, multicollinearity can be problematic if it occurs, and therefore we may find little or no overlap between explanatory variables (Petter et al. 2007; Henseler et al. 2009). In accordance with previous quantitative studies, we applied the reflective structure (Hair et al. 2016). Our PLS-SEM model was built up with two preparatory steps: (1) a target variable representing the food wastage of the respondent was composed with principal component analysis method; (2) the three attitude components were composed of a set of attitude variables that influenced the food wastage variable, as a result of factor analysis.

### Defining the target variable with principal component analysis

The aim of this study is to explore the behavioral reasons behind household food waste; therefore, we had to compose a target variable representing the food wastage level of certain consumers. In the survey, we examined the frequency of 16 potentially wasted food types with the help of a 1–5 Likert scale (higher number indicates higher frequency). For the target variable, only the 5 most frequently wasted food types were selected: meals (2.66), bakery products (2.48), dairy products (2.19), meat products (1.93), fresh fruits, and vegetables (1.72). The less frequently wasted food types did not deliver meaningful addition to this indicator, while significantly decreased the reliability of the combined variable.

The composed target variable was verified by Pearson correlation test (value of the coefficients were > 0.0001 in all cases) and KMO-Bartlett test (0.778, the acceptable range is > 0.6) (Osborne et al. 2008). The generated target variable explains 53% of the variance of the complete value set (Table 3).

**Table 3:** Explained variance by the generated target variable

Number of factors	Factor loading	Explained variance %	Cumulated explained variance %
1	2.666	53.316	53.316
2	0.839	16.772	70.087
3	0.601	12.010	82.098
4	0.497	9.938	92.035
5	0.398	7.965	100.000

### Description of the attitude components with factor analysis

**Fig. 1:**
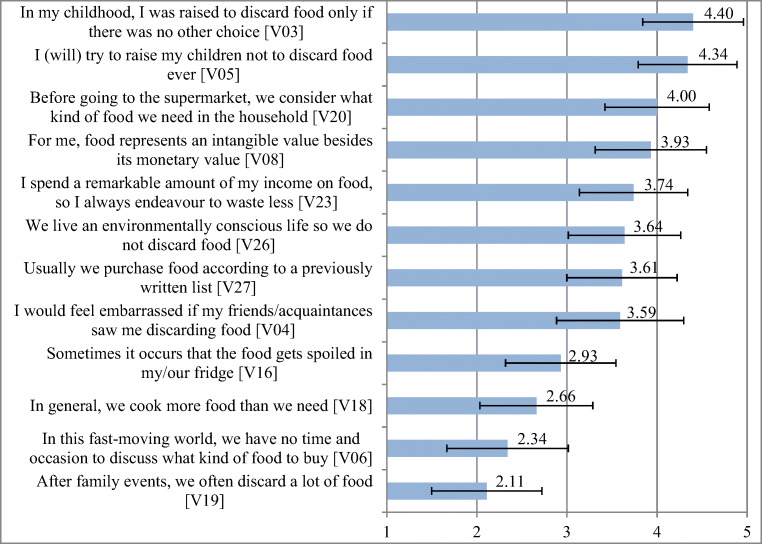
Descriptive results of attitude related questions (mean values of 1–5 Likert scale)

Several attitude-related variables were placed in the questionnaire to adequately support the 3-component attitude modeling (Fig. 1). During the factor analysis, a strong linear correlation could be detected among them (KMO = 0.819) (Table 8 in the Appendix). The generated factors contain 55% of the information content of the complete value set that can be considered to be satisfactory compared to the high number of explained variables (12).

Results of factor analysis can be interpreted on the multidimensional attitude theory. Based on the determinant variables, the identified factors include the following topics:

*Factor 1 – Affective elements*

- Emotional attitudes towards food (respect) (V08)
- Intrinsic motivation to prevent food waste generation (V04)
- Stigmatizing food waste as a social phenomenon (V05)
- Childhood emotional effects (V03)

*Factor 2 – Cognitive elements*

- Awareness of monetary damage to food waste (V23)
- Awareness of environmental problems associated with food waste (V26)
- Consciousness (V20)
- Planning and organization (V27)

*Factor 3 – Conative elements*

- Handling of food leftovers (V06)
- Food storage (V16)
- Cooking habits (V18)
- Meal as a family event/recreational activity (V19)

The composed component matrix with the factors is presented in Table 8 in the Appendix.

### Validation of reflective PLS-SEM model

The outer model was verified by the following process. Internal consistency was tested based on Cronbach’s alpha values and the Composition reliability values (Nunally and Bernstein 1994; Rossiter 2002; Hair et al. 2009; Henseler et al. 2015; Hair et al. 2016). We also tested the convergence of the structures, with respect to the outer loading values and the average variance extracted values (Hulland 1999; Hair et al. 2009, 2011, 2016). The differentiation criteria were also verified (Fornell and Larcker 1981; Hair et al. 2011). The test results are shown in Table 9 in the Appendix.

The inner model can be evaluated with bootstrap methods (beta values) in the aspect of the connection between latent structures and based on the predicted information (*R*^2^) (Davison and Hinkley 1997; Chin 1998; Tenenhaus et al. 2005; Hair et al. 2011; Hair et al. 2016). However, there are no minimum values accepted in the literature for this parameter, the graphical representation of the models contain the actual *R*^2^ values.

## Results and discussion

### Effect of demographical background

We have analyzed the relation between the target variable that represents food wastage and a set of demographical variables (age, sex, place of residence, region according to NUTS1 classification, education level, income status, level of awareness, and living with child under the age of 6) with variance analysis.

We have found statistically significant results in regard to age, income, education level, place of residence, and region (Table 4).

**Table 4:** Significant demographic group mean differences related to food wastage target variable values according to variance analysis (higher number means higher food wastage)

Age	Mean	Income	Mean	Education	Mean	Residence	Mean	Region	Mean
Under 30 years	0.415	Low	− 0.268	Primary school	0.055	Village	− 0.186	Central Hungary	0.212
30–39 years	0.372	Average	− 0.003	Vocational school	− 0.460	Town	− 0.051	Transdanubia	− 0.043
40–59 years	− 0.045	High	0.315	High school graduation	− 0.048	Capital city	0.261	Great Plain and North	− 0.130
Over 60 years	− 0.553			Higher education	0.094				

*p* < 0.05 (95% CI)

Although statistically significant differences in regard of sex, level of awareness, and living with child under 6 years of age could not be detected, our results still indicate a likely relation between these variables and food wastage that may be validated by the increase of the sample size (Table 5).

**Table 5:** Not significant demographic group mean differences related to food wastage target variable values according to variance analysis (higher number means higher food wastage)

Sex	Mean	Level of awareness	Mean	Child under 6 years	Mean
Women	− 0.0344	Low	0.0752	Yes	0.0948
Men	0.0393	Average	− 0.0292	No	− 0.0004
		High	− 0.0331		

*p* < 0.05 (95% CI)

Consumers’ level of awareness regarding food labeling and storing was measured by true or false questions about best before and use by dates, proper temperature of the fridge, and storing of different types of food*.*

### PLS-SEM behavior modeling

Applying the PLS-SEM method, our study confirmed the previous finding that consumer food wastage is not a result of a single behavior but is influenced by a combination of several behavioral patterns (Mondéjar-Jiménez et al. 2016).

**Fig. 2:**
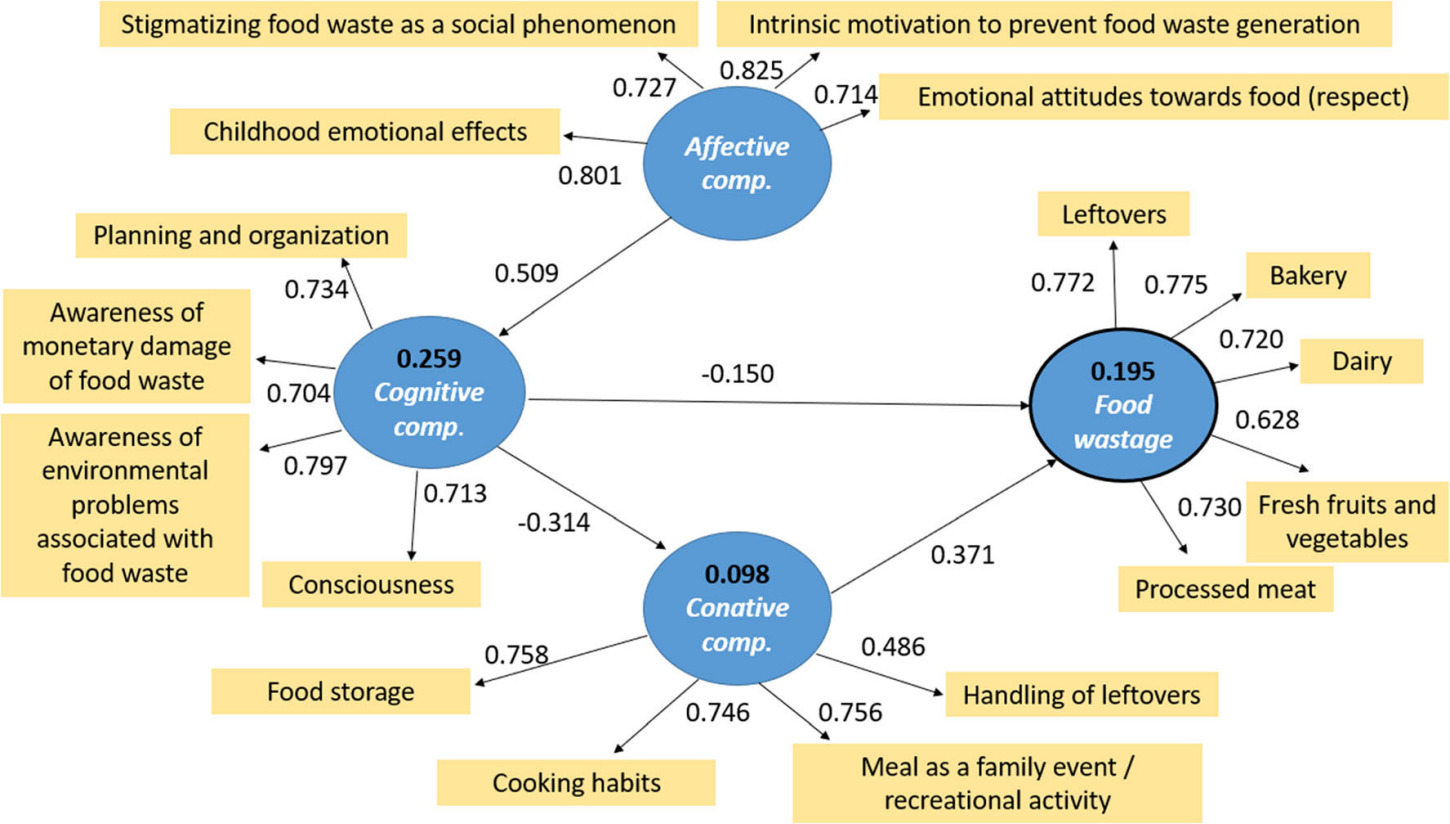
Normative PLS-SEM model of consumer food waste behavior

The *normative* PLS-SEM model has revealed that food wastage is affected by the cognitive and the conative attitude components directly (endogen components of the SEM model), and influenced indirectly by the affective attitudes (exogen component of the model) (Fig. 2).

The standard errors were bootstrapped by considering 5000 sub-samples. The results of direct structural relationships are statistically significant at 5% level (*p* value < 0.01). Based on the mathematical proof of the normative PLS-SEM model, we justified that the behavior of Hungarian consumers towards food waste is determined by the affective, cognitive, and conative attitude components, although their influences are different (Table 6). The conative component has the strongest direct influence (0.371) to the wastage target value. The affective component has an indirect effect, but it still has a significant direct and indirect contribution to the forming of food wasting consumer behavior.

**Table 6:** Value of aggregated effects of the attitude components to food wastage behavior derived from the normative PLS-SEM model

Latent structures	Affective component	Cognitive component	Conative component	Wastage
Affective component		0.509	− 0.160	− 0.135
Cognitive component			− 0.314	− 0.266
Conative component				0.371

**Fig. 3:**
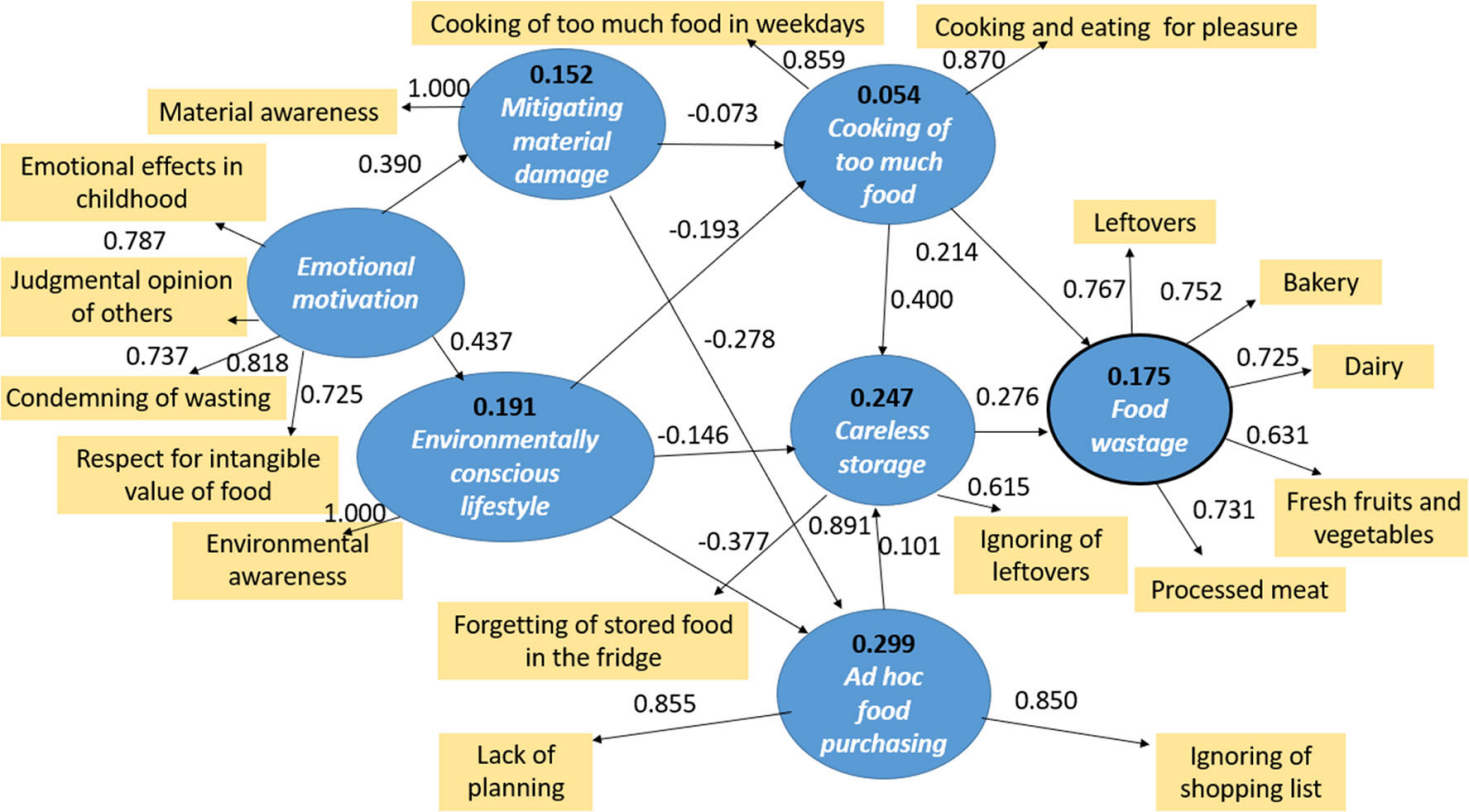
Explicative model of consumer food waste behavior

Although the *normative* PLS-SEM model gives an indication about the role of the different attitudes that can be targeted during an awareness raising campaign, communication actions usually need more definite information to construct effective messages. For this reason, by using the same set of variables that the normative model was constructed of, we have created an *explicative* model also. In this model, we paid attention to those variables that deliver more practical information to communication experts (Fig. 3).

The standard errors were bootstrapped by considering 5000 sub-samples. The results of direct structural relationships are statistically significant at 5% level (*p* value < 0.01). Through the explicative model, we proved the existence of such latent structures that are important from a practical point of view and affect household food waste either directly or indirectly. These factors show a tight correlation—even on their own—with the extent of waste (Table 7).

**Table 7:** Value of aggregated effects of latent structures to food wastage behavior derived from the explicative PLS-SEM model

Latent structures	Wastage
Cooking of too much food	0.314
Careless storage	0.264
Environmentally conscious lifestyle	− 0.132
Ad hoc food purchasing	0.086
Emotional motivation	− 0.076
Mitigating material damage	− 0.047

The phenomenon of cooking too much food had the strongest correlation with food waste generation. This result closely correlates to the fact that leftovers of home-cooked meals found to be the most frequently wasted food type in this study and our previous investigation on food waste composition (Szabó-Bódi et al. 2018) as well. This observation can be generalized to other countries also, based on recent waste composition studies (Hanssen et al. 2016; Djekic et al. 2019). Careless food storage was also an observed latent structure that contributed to wastage. Our research did not reveal significant correlation between unplanned food purchase and the extent of food waste, which is in contrast with the results of certain other studies (Jörissen et al. 2015; Stefan et al. 2013). Jörissen et al. (2015) asked respondents whether they used a shopping list and compared the answers directly to the amount of generated food waste. Stefan et al. (2013) by a structural modeling method found that a significant contributing factor of food waste is bad planning routines. However, they did not discuss other possible behavioral patterns, such as storing habits or environmentally consciousness, which could have revealed further links to food wastage. However, the PLS-SEM method used in the present study was suitable for exploring complex casual relationships between the wide range of variables that the questionnaire contained. The results suggest that unplanned food shopping contributes to food waste generation, but only indirectly. This could be explained if different factors of food wastage were classified as “reasons”—direct effects—or “drivers”—indirect triggering effects (Jörissen et al. 2015). Likewise, Visschers et al. (2016) found that household planning habits were not related directly to food waste, only correlated with consumers’ intention to avoid wasting. Environmentally conscious lifestyle, emotional motivation, and mitigating material damage proved to be indirect contributors too. As the model indicates, emotional motivation correlates to childhood emotional effects and judgment of others.

## Conclusion

In this study, we summarized the research results that served to plan a national level household food waste prevention campaign that considers behavior insights. The findings of the normative and explicative PLS-SEM models were utilized in the Hungarian *Wasteless/Maradék nélkül* program. Key attitudes and behavioral elements identified by the research were targeted in communication actions and the key messages together with the relevant knowledge background later became part of the Hungarian primary school education.

The normative PLS-SEM model of consumer food waste behavior provided evidence that conative behavioral components should be taken into consideration primarily during the design of prevention campaigns. This indicates that childhood education and awareness raising should be key activities, because they deliver impulses before routines that lead to excessive food waste would have been rooted too deep in the individual behavioral pattern. Cognitive attitude elements—supported significantly by affective elements—also seem to have impact on food waste behavior to some extent. This provides a recipe how to address this issue in communication with adult consumers: a mixture of emotional and rational messages is recommended. The explicative PLS-SEM model further develops this issue by identifying the critical points in consumer behavior. It is clear that preparing excessive amount of food is responsible for the majority of food waste in households. Second to that, careless food storage was identified as an important intervention area for the food waste preventive campaign. Unplanned or impulse purchase of food plays a less significant role according to our results. It is still important to mention as a relevant issue, because it clearly contributes to careless food storage, as our model indicates. According to this, the seeming controversy between our findings and other studies might be also explained: impulse shopping itself does not generate excess food waste if the household pays attention to proper storage and timely consumption of food stuffs that were purchased unplanned.

Environmentally conscious lifestyle, appearing as a latent structure of the explicative model is the most efficient preventive factor in the formulation of consumer behavior in regard to food waste, with an effect that greatly exceeds the preventive strength of the mitigation of material losses. The role of childhood emotional effects underlines the need for school programs again, while the observation in regard to “judgment of others” projects that convincing opinion leaders to show good examples in field of food waste prevention would result in a multiplicative effect. This result provides justification to the Recommendations for Action in Food Waste Prevention, developed by the EU Platform on Food Losses and Food Waste (2019) that suggests that all food chain actors should work for “promoting value of food and working to shift social norms so that wasting food is no longer socially acceptable”.

## Appendix

**Table 8:** Composed factor matrix from the attitude-related questions

Variable	Factors		
	1 (explained variance: 20.088%)	2 (explained variance: 17.877%)	3 (explained variance: 16.961%)
In my childhood, I was raised to discard food only if there was no other choice (V03)	0.779	0.178	− 0.040
I would feel embarrassed if my friends/acquaintances saw me discarding food (V04)	0.706	0.117	− 0.071
I (will) try to raise my children not to discard food ever (V05)	0.825	0.146	− 0.072
For me, food represents an intangible value besides its monetary value (V08)	0.597	0.274	− 0.054
I spend a remarkable amount of my income on food, so I always endeavor to waste less (V23)	0.203	0.660	− 0.065
We live an environmentally conscious life so we do not discard food (V26)	0.323	0.590	− 0.266
Usually we purchase food according to a previously written list (V27)	0.060	0.809	− 0.073
Before going to the supermarket, we consider what kind of food we need in the household (V20)	0.220	0.708	− 0.150
In this fast-moving world, we have no time and occasion to discuss what kind of food to buy (V06)	− 0.233	− 0.063	0.480
Sometimes it occurs that the food gets spoiled in my/our fridge (V16)	0.019	− 0.219	0.656
In general, we cook more food than we need (V18)	0.067	− 0.107	0.780
After family events, we often discard a lot of food (V19)	− 0.110	− 0.033	0.805

**Table 9:** Validation criteria of PLS-SEM model

Tested criteria	Value	Result
Internal consistency	Cronbach’s alpha values	> 0.6 except in case of one component
	Composition reliability	> 0.6 in all cases
Convergence of structures	Outer loading	> 0.4 in all cases
	Average variance extracted	> 0.5 except in case of 1 component
Differentiation criteria	Fornell-Larcker test	Passed
	Cross-loading coefficient	Passed
